# Performance du GeneXpert MTB/RIF^®^ dans le diagnostic de la tuberculose extra-pulmonaire à Dakar: 2010-2015

**DOI:** 10.11604/pamj.2016.25.129.10065

**Published:** 2016-11-02

**Authors:** Awa Ba Diallo, Abdoulkader Issifi Kollo, Makhtar Camara, Seynabou Lo, Gedeon Walbang Ossoga, Moustapha Mbow, Farba Karam, Mame Yacine Fall Niang, Aliou Thiam, Awa Ndiaye Diawara, Souleymane Mboup, Aissatou Gaye Diallo

**Affiliations:** 1 Laboratoire de Bactériologie, Virologie du CHNU Aristide Le Dantec, Sénégal; 2 Département des Sciences Biologiques, Université Cheikh Anta Diop, Dakar, Sénégal; 3 UFR Sciences de la Santé, Université Gaston Berger, Saint Louis

**Keywords:** GeneXpert MTB/RIF, tuberculose extra-pulmonaire, Dakar, GeneXpert MTB/RIF, extra-pulmonary tuberculosis, Dakar

## Abstract

**Introduction:**

Le défi des pays en voie de développement est la disponibilité de méthodes de diagnostic rapide et précis pour le management de la tuberculose. Des techniques moléculaires offrent cet avantage et nous avons utilisé le test GeneXpert MTB/RIF dans le diagnostic de la tuberculose extra-pulmonaire pour évaluer sa performance par rapport aux méthodes conventionnelles.

**Méthodes:**

Entre 2010 et 2015, 544 échantillons cliniques extra-pulmonaires ont été recueillis et traitées par la microscopie, la culture et le GeneXpert. L'étude de la sensibilité aux antituberculeux a été effectué avec le MGIT 960. Le Génotype MTBDR*plus* a été utilisé pour confirmer les cas de résistance à la rifampicine détectés par le système GX**.**

**Résultats:**

La population d'étude de 544 patients incluait 55,15% d'hommes et 44,85% de femmes. L'âge des patients variait entre 1 à 92 avec la majorité dans le groupe d'âge 18-45 ans. La sensibilité et la spécificité globale de la microscopie étaient de 43,86% et 98,36%, et pour le GeneXpert^®^ 94,74% et 97,95% respectivement avec 95% IC. Deux résultats de résistance à la rifampicine discordants ont été trouvées entre le test GeneXpert et la méthode phénotypique. Les résultats du test MTBDR*plus* ont montré une concordance de 100% avec ceux du MGIT 960 pour les cas discordants de résistance à la rifampicine.

**Conclusion:**

Cette étude a montré que le test GeneXpert a une plus grande sensibilité pour le diagnostic de routine de la tuberculose extra-pulmonaire et devrait être utilisé à la place de la microscopie. Les cas de résistance à la rifampicine détectés par le GeneXpert doivent être confirmés par d'autres tests moléculaires avant d'initier un traitement.

## Abstract (en)

**Introduction:**

The challenge facing developing countries is the availability of methods for rapid and accurate diagnosis of tuberculosis. Some molecular techniques offer this advantage, so we used GeneXpert MTB / RIF test in the diagnosis of extra-pulmonary tuberculosis to evaluate its performance compared with conventional methods.

**Methods:**

Between 2010 and 2015, 544 extrapulmonary clinical specimens were collected and analyzed by microscopy, culture and GeneXpert. The evaluation of antitubercular susceptibility testing was performed using the MGIT 960 system. Genotype MTBDRplus was used to confirm the cases of rifampicin resistance detected by the GX system.

**Results:**

The study population included 544 patients, 55.15% men and 44.85% women. Patients age ranged from 1-92 years with the majority in the 18-45 age group. The sensitivity and the overall specificity of microscopy was 43.86% and 98.36%, 94.74% and 97.95% for GeneXpert^®^ respectively (95% CI). There were two discrepant rifampicin-resistant results between GeneXpert test and phenotypic method. Among these cases MTBDRplus test results showed 100% agreement with those of the MGIT 960.

**Conclusion:**

This study shows that the GeneXpert test exhibits high sensitivity for routine diagnosis of extra-pulmonary tuberculosis and should be used instead of microscopy. The cases of rifampicin resistance detected by GeneXpert should be confirmed by other molecular testing methods before initiating treatment.

## Introduction

La tuberculose (TB) reste encore l'une des maladies infectieuses causant le plus de décès dans le monde. En effet, en 2014 selon l'Organisation mondiale de la santé (OMS), 9,6 millions de personnes auraient contractée la maladie avec 1,5 million de cas de décès dont 400 000 personnes vivant avec le virus de l'immunodéficience humaine (VIH) [1]. Sur les 9,6 millions de cas de TB, 28% vivaient dans la région africaine avec le plus grand nombre de nouveaux cas détectés. Au Sénégal, 13647 cas de TB ont été déclarés parmi lesquels on dénombre 1653 cas de tuberculose extra-pulmonaire (TBEP) en 2014 [2]. Un regain d'intérêt est observé vis-à-vis de ces formes extra pulmonaires de la TB du fait de la complexité de leur diagnostic et de l'augmentation relative des cas enregistrés durant ces dernières années. En effet, le Sénégal a enregistré progressivement 1366 cas de TBEP en 2011, 1524 cas en 2012, puis 1618 cas en 2013. Pourtant le diagnostic de la TBEP due aux espèces du complexe *Mycobacterium tuberculosis* (MTBC) reste difficile à établir en raison de la faible quantité de mycobactéries présentes dans les échantillons cliniques comparée à celle observée dans les cas d'infections pulmonaires [3]. Cette faible quantité explique la difficulté du diagnostic rapide de la TBEP par les méthodes conventionnelles disponibles dans les pays en voie de développement. Pour répondre au besoin urgent de mettre en place un outil de diagnostic performant et rapide, un test moléculaire entièrement automatisé pour la détection de MTBC ainsi que la résistance à la rifampicine (RIF) a été mis au point dans le cadre d'un partenariat public-privé [4]. En décembre 2010, l'OMS a approuvé le GeneXpert MTB/RIF (GX) (Cepheid, Sunnyvale, CA, USA) pour le diagnostic de première intention de la TB pulmonaire chez les patients vivants avec le VIH ou suspectés de développer une TB multi résistante [5]. Quelques travaux comme ceux de Tortoli et al ont conclu à la validation clinique de cet automate pour le diagnostic de la TBEP avec une sensibilité et une spécificité respectivement de 81,3% et 99,8% [6]. Cependant peu d'études recommandent son utilisation en cas de suspicion de TBEP [7]. Par ailleurs aucune donnée n'est disponible sur la performance du GX dans le diagnostic de la TBEP en Afrique de l'Ouest. Le but de cette étude était d'évaluer la performance du GX dans le diagnostic de la TBEP en utilisant la culture comme méthode de référence.

## Méthodes

### Cadre et population d'étude

**Figure 1:**
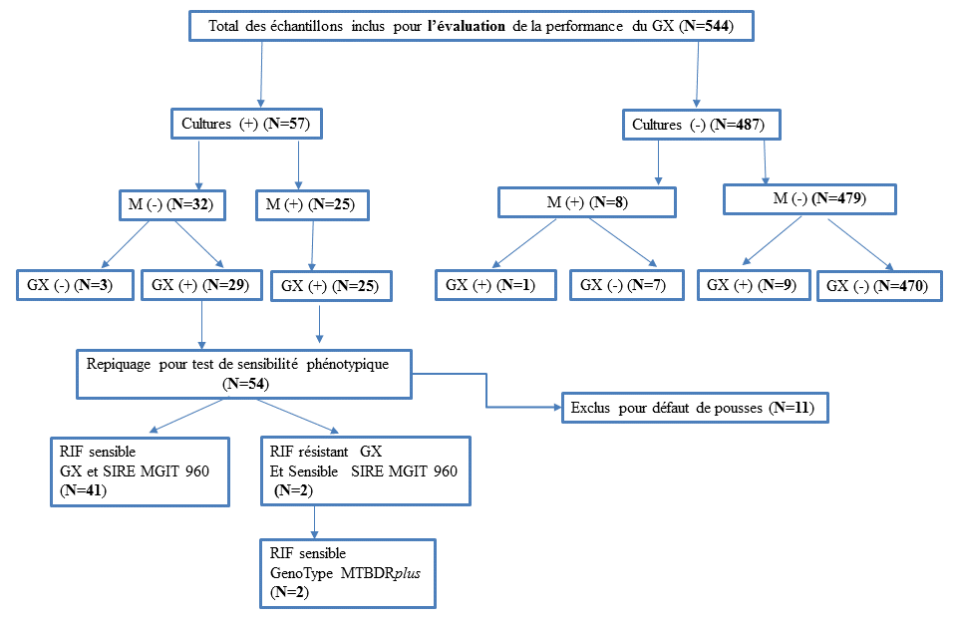
Caractéristiques des résultats des échantillons de l’étude suivant les techniques utilisées

C'est une étude rétro-prospective avec des échantillons collectés entre Janvier 2010 et décembre 2015 et portant sur 566 cas de patients suspects d'avoir une tuberculose extra- pulmonaire. Parmi les 566 patients enrôlés dans l'étude, 14 ont été exclus parce que la culture, la microscopie et le test TBcID des prélèvements ont permis de mettre en évidence la présence de mycobactéries non tuberculeuses. Huit autres échantillons ont été exclus pour cause d'informations démographiques manquantes sur les patients concernés. Les 544 échantillons restants ont été recueillis chez des patients qui provenaient essentiellement de la région de Dakar et de sa périphérie. La Figure 1 détaille les échantillons inclus dans l'étude et les techniques utilisés. L'étude a été effectuée dans l'unité de Mycobactériologie du laboratoire de Bactériologie-Virologie du Centre Hospitalier National Universitaire (CHNU) Aristide Le Dantec de Dakar au Sénégal. Un échantillon clinique suivant le site de localisation a été recueilli pour chaque patient puis analysé. Les échantillons cliniques étaient composés de : liquide céphalorachidien (LCR), liquide d'épanchement (liquide d'ascite (LA), liquide pleural (LP), liquide péritonéal (LPT), liquide péricardique (LPC), de plus, d'urines et autres types d'échantillons composés de biopsie, sperme, prélèvement urétral, moelle osseuse, liquide de dialysat, et autres liquides biologiques dont les origines n'ont pas été précisées. Les autres types d'échantillons ont été classés dans le groupe autre liquide d'épanchement (ALE). Les échantillons extra-pulmonaires inclus dans cette étude ont été si nécessaires décontaminés selon le protocole décris par Kubica et al [8]. Des frottis ont été préparés à partir des culots de centrifugation pour chaque échantillon, colorés par la méthode de Ziehl-Neelsen et observés au microscope optique.

### Test GeneXpert MTB/RIF

Le GX est un système basé sur la réaction de polymérisation en chaîne (PCR) en temps réel qui détecte l'ADN de MTBC et les mutations qui confèrent la résistance à la RIF en moins de 120 minutes à partir d'échantillons cliniques [9]. Pour détecter les souches du complexe MTB et les mutations associées à la résistance à la RIF, la région centrale cible de 81 pb du gène rpoB est amplifiée et couplée avec cinq balises moléculaires ou probes (A, B, C, D, E de type « beacons ») qui se chevauchent le long de la séquence cible [4, 10]. Les culots de décontamination ont été testés au GX suivants les instructions du fabricant [11].

### Culture des mycobactéries

Pour chaque culot de décontamination, 0,5 ml a été inoculé dans des tubes MGIT contenant 7 ml de milieu 7H9 Middlebrook additionné de 0,8 ml de supplément de croissance BD PANTA puis incubés dans l'automate. Une culture sur milieu solide Lowenstein-Jensen (LJ) a été faite en parallèle avec 100 µl de culot puis incubés à 37^°^C. Les cultures positives ont été confirmées par la microscopie après coloration de Ziehl-Neelsen et avec le test TBcID de Becton Dickinson (BD) qui détecte l'antigène MPT64 spécifique du complexe M. tuberculosis selon le protocole spécifié par le fabricant [12].

### Détermination de la résistance phénotypique

L'étude de la sensibilité aux molécules antituberculeuses (Streptomycine, Isoniazide, Rifampicine et Ethambutol) a été effectuée sur les cultures positives aux souches du complexe MTB. Le protocole Bactec MGIT 960 de BD a été utilisé avec des concentrations d'antibiotiques (ATB) critiques de1µg/ml, 0,1 µg/ml, 1µg/ml et 5µg/ml respectivement pour la Streptomycine (STR), l'Isoniazide (INH), la Rifampicine (RIF) et l'Ethambutol (EMB) (méthode SIRE MGIT 960).

### Extraction de l'ADN et détermination de la résistance génotypique

À partir d'une culture positive sur milieu solide, des colonies ont été prélevées, suspendues dans du tampon Tris-Acide éthylène diamine tétra acétique (Tris-EDTA) puis inactivées à 99^°^C. Le reste de la procédure a été effectué par la méthode au Cétyl trimethyl ammonium bromide (CTAB) selon le protocole décrit par van Soolingen et al [13]. Le test de détection de la résistance moléculaire à la RIF et à l'INH a été effectué en utilisant le kit Genotype MTBDRplus version 2.0 de Hain lifescience pour les cas de résistance à la RIF détectés par le GX. Le test a été effectué selon le protocole fourni par le fabricant [14].

### Analyses statistiques

**Figure 2:**
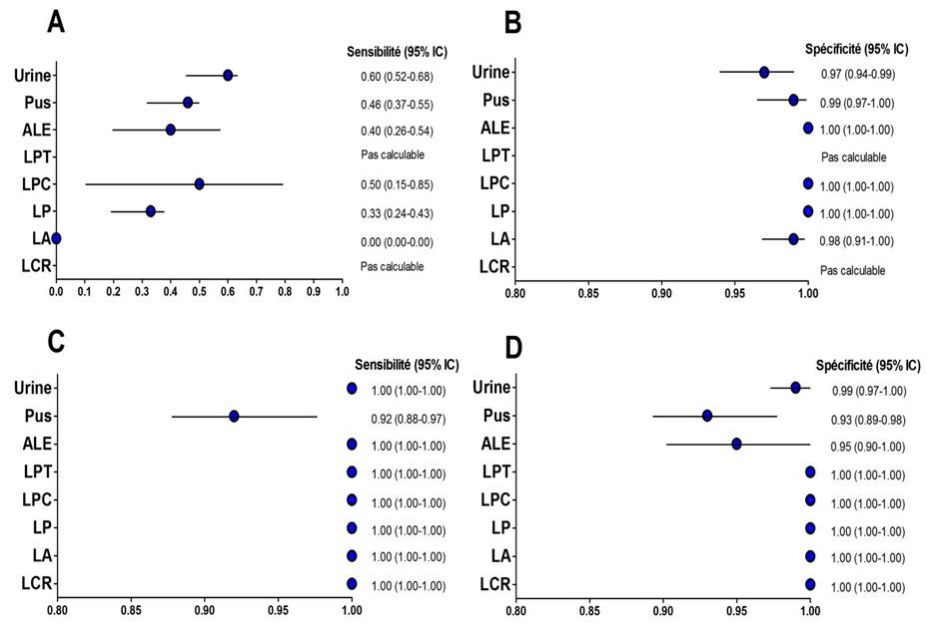
Forest plot des sensibilités, spécificités du GeneXpert et de la microscopie par type d’échantillons

Les données ont été saisies avec le logiciel de calcul Microsoft Excel 2007 et introduites dans la base de données du logiciel Epi Info^TM^6 (Centers for Disease Control and Prevention, Atlanta, GA, Etats-Unis). Les calculs statistiques ont été effectués avec le programme « EPITABLE calculateur » du logiciel. Les graphes de la Figure 2 ont été effectués par le logiciel GraphPad Prism version 5.00 pour Windows (GraphPad Software Inc.). Tous les calculs statistiques ont été effectués en fixant l'intervalle de confiance (IC) à 95%.

## Résultats

Parmi les 544 patients, 61% étaient hospitalisés dans divers services cliniques du CHNU et 39% provenaient d'autres structures de santé externes. 55% des patients étaient de sexe masculin et 45% de sexe féminin. Les patients de moins de 18 ans représentaient 29,8%, ceux entre 18 et 45 ans 38,9% en enfin ceux ayant plus de 45 ans 31,3%. Les échantillons cliniques inclus dans l'étude étaient composés de LCR (n=17), de LA (n=79), de LP (n=99), de LPC (n=8), de LPT (n=5), de pus (n=129), d'urines (n=157) et d'ALE (n=50). Les résultats des différents types d'analyses effectués sont résumés sur la Figure 1. Les sensibilités, spécificités valeurs prédictives positives (VPP) et négatives (VPN) portant sur la détection des souches du MTBC sont détaillées dans le Tableau 1. Par ailleurs les résultats ont révélé des sensibilités et des spécificités variables selon la nature de l'échantillon testé voir Figure 2.

**Tableau 1:** Performance du GeneXpert MTB/RIF et de la microscopie

	% Sensibilité (IC 95%)	% Spécificité (IC 95%)	%VPP (IC 95%)	%VPN (IC 95%)
**Microscopie**				
Tous les échantillons	43,86 (39,69-48,03)	98,36 (97,29-99,43)	75,76 (72,16-79,36)	93,74 (91,70-95,77)
**GeneXpert**				
Tous les échantillons	94,74 (92,86-96,61)	97,95 (96,75-99,14)	84,38 (81,32-7,43)	99,38(98,71-100,04)
M (+)	96,15 (89,59-102,72)	100 (100-100)	100 (100-100)	87,50 (76,22-98,78)
M (-)	87,50 (76,22-98,78)	99,37 (98,68-100,05)	90,63 (88,10-93,15)	98,12 (96,94-99,30)

**IC 95%=** intervalle de confiance à 95%; **VPP=** valeur prédictive positive; **VPN=** valeur prédictive négative; **M (+)=** microscopie positive; **M (-)=** microscopie négative

Concernant la détection de la résistance à la RIF, parmi les 54 échantillons dont la culture et le GX ont donné des résultats positifs, 20% (N=11) n'ont pas pu être testés pour défaut de pousse lors du repiquage à partir des culots conservés. Les souches MTBC issues de 43 échantillons ayant donné des pousses, étaient tous sensibles à la RIF par la méthode SIRE MGIT 960 tandis que le GX a détecté 95,35% (41/43) de sensibilité à la RIF. Cependant, deux cas de discordance entre le test GX et le test de résistance phénotypique à la RIF utilisant la technique SIRE MGIT 960 ont été trouvés (Tableau 2). Pour ces deux cas de discordance, le test Genotype MTBDRplus a donné des résultats à 100% en accord avec ceux obtenus par le système SIRE MGIT 960.

**Tableau 2:** Profil de résistance à la Rifampicine par les techniques SIRE MGIT 960, GeneXpert MTB/RIF et GenoType MTBDRplus pour les deux cas discordants

Identifiant échantillon clinique	SIRE MGIT 960	GeneXpert (niveau de détection, mutations)	GenoType MTBDRplus (Gène cible)
12/SEN 579 LPC	SENSIBLE	RESISTANT (Very low, probe A&E)	SENSIBLE (Gène rpoB sauvage)
13/SEN 266 Pus	SENSIBLE	RESISTANT (Very low, probe D)	SENSIBLE (Gène rpoB sauvage)

**IC 95%=** intervalle de confiance à 95%; **VPP=** valeur prédictive positive; **VPN=** valeur prédictive négative; **M (+)=** microscopie positive; **M (-)=** microscopie négative

## Discussion

La sensibilité et la spécificité du GX dans le diagnostic de la tuberculose pulmonaire et extra pulmonaire ont été évaluées dans plusieurs études avec néanmoins des résultats variables [3, 6, 7, 15–18].Les résultats de notre étude ont révélé que pour tous les échantillons inclus, le GX a une sensibilité de 94,74% (54/57) contrairement à la microscopie qui n'a détecté que 43,86% (25/57)des souches de MTBC. Ce qui fait du GX un outil plus performant que la microscopie dans le diagnostic de la TBEP comme dans les études de Tortoli et al et d'Iram et al [6, 19]. La sensibilité du GX parmi les échantillons testés M (+) était de 96.15% tandis que celle obtenue pour les échantillons testés M (-) était de 87,5%. Ces valeurs indicatives de la performance du GX sont approximativement les mêmes que celles obtenus par l'auteur Vadwai et al [20]. Les résultats de la performance du GX et de la microscopie s'expliqueraient en partie par le fait que les limites de détection de la culture et du GeneXpert seraient respectivement de 10-100 CFU/ml et de 131 CFU/ ml, alors que celles de la microscopie seraient comprises entre 5000 et 10000 CFU/ ml [21].

Cependant la sensibilité et la spécificité du GX a aussi montré des variations selon la nature de l'échantillon clinique analysé. Dans cette présente étude, la sensibilité du GX pour les LCR, LA, LP, LPC, LPT, ALE et les urines est de 100% pour chaque type d'échantillon sauf les pus pour lesquels on a obtenu une sensibilité de 92%. Ces résultats confirment la bonne performance du GX dans le diagnostic de la TBEP malgré la nature pauci bacillaire des échantillons cliniques extrapulmonaires. L'étude de Vadwai et al a révélé des sensibilités de 100%, 100%, 94% pour les LPC, LPT et pus respectivement [20] tandis que celle de Hillemann et al a donné une sensibilité de 100% pour les urines [3]. Par contre notre étude a révélé, notamment pour les LCR, LP et LA, des sensibilités plus élevées comparées à celles obtenues dans les études d'auteurs comme Denkinger et al et Sharma et al [22, 23]. Cette variation de la sensibilité et de la spécificité du test GX selon le type de prélèvement s'expliquerait par le fait que la charge mycobactérienne qui est variable selon les différents compartiments du corps soit le principal déterminant de la positivité du test GX [24].

L'automate GX a montré que 95,34% des échantillons cliniques analysés étaient sensibles à la Rifampicine. Ces mêmes échantillons étaient tous sensibles à la RIF par la méthode phénotypique SIRE MGIT 960, ce qui implique une parfaite concordance entre le GX et la technique SIRE MGIT 960 pour les souches sensibles à la RIF. Les travaux d'autres auteurs ont abouti à la même conclusion [25, 26]. Cependant pour les deux cas de discordance observés entre le GX et la technique SIRE MGIT 960, la technique MTBDR*plus* a confirmé les résultats obtenus par la méthode phénotypique à savoir la sensibilité de ces deux souches à la RIF. En effectuant le séquençage du gène *rpo*B (référence en matière de détection de la résistance génotypique à la RIF), l'étude de Williamson et al a montré que le GX était capable de détecter des souches faussement résistantes à la RIF [27]. Les travaux d'Ocheretina et al ont montré qu'une grande partie des souches faussement résistantes à la RIF étaient détectées en faible quantité “Very low”' par le GX [28]. Ce type de résultats obtenus par le GX pourrait inciter les cliniciens à prescrire aux patients un traitement inadapté en considérant que les souches responsables de l'infection seraient résistantes à la RIF. Ce qui impliquerait une administration abusive des antituberculeux de seconde ligne dont l'utilisation est réglementée par des normes strictes en vue d'éviter l'émergence de souches ultra résistantes. Ces deux cas de discordance obtenus dans notre étude réconforteraient les conclusions du “the Centers for Disease Control and Prevention” (CDC) qui recommanderait la confirmation des cas de résistance à la RIF détectés par le test GX [29] avant l'initiation de tout traitement antituberculeux.

## Conclusion

Cette étude a montré que le test GX est plus performant que les méthodes conventionnelles de diagnostic comme la microscopie qui est utilisée en routine dans les pays en voie de développement pour le diagnostic de la tuberculose pulmonaire et extra pulmonaire. Les sensibilités et spécificités obtenues font du GX un outil utile et rapide pour le diagnostic de première intention de la TBEP dans notre laboratoire. Cependant malgré sa bonne performance, les cas de résistance à la RIF détectés par le GX devraient être confirmés par d'autres techniques moléculaires plus efficaces comme le séquençage avant l'initiation de tout traitement antituberculeux.

### Etat des connaissances actuelle sur le sujet

- La grande difficulté du diagnostic de la tuberculose extra pulmonaire par les méthodes conventionnelles utilisées dans les pays en voie de développement;
- Le test GeneXpert MTB/RIF^®^ est performant dans le diagnostic de la TBEP dans certaines régions du monde;
- Il n'existe aucune donnée sur la performance du GeneXpert MTB/RIF^®^ en Afrique de l'ouest.

### Contribution de notre étude à la connaissance

- Notre étude contribue à l'amélioration de la disponibilité de données sur la performance du GeneXpert MTB/RIF^®^ en Afrique de l'ouest et au Sénégal en particuliers.
